# Improvement in White Matter Tract Reconstruction with Constrained Spherical Deconvolution and Track Density Mapping in Low Angular Resolution Data: A Pediatric Study and Literature Review

**DOI:** 10.3389/fped.2017.00182

**Published:** 2017-08-30

**Authors:** Benedetta Toselli, Domenico Tortora, Mariasavina Severino, Gabriele Arnulfo, Andrea Canessa, Giovanni Morana, Andrea Rossi, Marco Massimo Fato

**Affiliations:** ^1^Department of Informatics, Bioengineering, Robotics and System Engineering (DIBRIS), University of Genoa, Genoa, Italy; ^2^Neuroradiology Unit, Istituto Giannina Gaslini, Genoa, Italy

**Keywords:** constrained spherical deconvolution, tractography, brain imaging, children, neonates, probabilistic tractography, 1.5 T, track density imaging

## Abstract

**Introduction:**

Diffusion-weighted magnetic resonance imaging (DW-MRI) allows noninvasive investigation of brain structure *in vivo*. Diffusion tensor imaging (DTI) is a frequently used application of DW-MRI that assumes a single main diffusion direction per voxel, and is therefore not well suited for reconstructing crossing fiber tracts. Among the solutions developed to overcome this problem, constrained spherical deconvolution with probabilistic tractography (CSD-PT) has provided superior quality results in clinical settings on adult subjects; however, it requires particular acquisition parameters and long sequences, which may limit clinical usage in the pediatric age group. The aim of this work was to compare the results of DTI with those of track density imaging (TDI) maps and CSD-PT on data from neonates and children, acquired with low angular resolution and low b-value diffusion sequences commonly used in pediatric clinical MRI examinations.

**Materials and methods:**

We analyzed DW-MRI studies of 50 children (eight neonates aged 3–28 days, 20 infants aged 1–8 months, and 22 children aged 2–17 years) acquired on a 1.5 T Philips scanner using 34 gradient directions and a b-value of 1,000 s/mm^2^. Other sequence parameters included 60 axial slices; acquisition matrix, 128 × 128; average scan time, 5:34 min; voxel size, 1.75 mm × 1.75 mm × 2 mm; one b = 0 image. For each subject, we computed principal eigenvector (EV) maps and directionally encoded color TDI maps (DEC-TDI maps) from whole-brain tractograms obtained with CSD-PT; the cerebellar-thalamic, corticopontocerebellar, and corticospinal tracts were reconstructed using both CSD-PT and DTI. Results were compared by two neuroradiologists using a 5-point qualitative score.

**Results:**

The DEC-TDI maps obtained presented higher anatomical detail than EV maps, as assessed by visual inspection. In all subjects, white matter (WM) tracts were successfully reconstructed using both tractography methodologies. The mean qualitative scores of all tracts obtained with CSD-PT were significantly higher than those obtained with DTI (*p*-value < 0.05 for all comparisons).

**Conclusion:**

CSD-PT can be successfully applied to DW-MRI studies acquired at 1.5 T with acquisition parameters adapted for pediatric subjects, thus providing TDI maps with greater anatomical detail. This methodology yields satisfactory results for clinical purposes in the pediatric age group.

## 1 Introduction

Diffusion-weighted magnetic resonance imaging (DW-MRI) has become the method of choice to noninvasively analyze brain structure and pathology in the clinical setting, providing *in vivo* quantitative and qualitative information about white matter (WM) microstructure and fiber tract pathways (see for example Schaefer et al. (1) for a review of the possible applications, or Moritani et al. (2) for a more complete discussion). This analysis is performed by mapping the motion of water molecules in the brain; while in the cerebrospinal fluid and gray matter, water motion does not significantly prevail in any direction (i.e., isotropic diffusion); in WM, water molecules move preferentially along the direction of axons and fiber tracts (i.e., anisotropic diffusion). The patterns followed by the water thus reflect the underlying tissue structure, allowing the tracing and reconstruction of WM fiber tracts. The sensitization of the image to diffusion is obtained by varying the homogeneity of the magnetic field with gradient pulses applied in several directions.

Historically, the first method to be developed for analyzing DWI sequences was Diffusion Tensor Imaging (DTI) (3): this method estimates a diffusion tensor for each voxel, from which the main diffusion direction is computed as the major eigenvector of the tensor. DTI yields good quantitative results and is routinely applied in clinical settings to compute diffusion metrics: six gradient directions are sufficient for the estimation of the tensors and to obtain scalar diffusion maps. However, since DTI computes a single main diffusion direction per voxel, it cannot correctly reconstruct those voxels in which two or more fiber tracts cross or are arranged in more complex configurations, nor can cortical terminations of the fibers be precisely determined (4). In order to overcome this limitation, several methods were developed (see for example Schaefer et al. (5) for a review). In particular, Constrained Spherical Deconvolution (CSD) has been widely applied to advanced diffusion studies in adult subjects with excellent results (6, 7). More recently, a further step in DWI post processing derived by whole-brain probabilistic streamline tractography, the track density imaging (TDI) (8), has enabled exploring the anatomy of human brain in greater details, allowing to compute maps in which the visualization of dense fiber tracts is improved by interpolating information from each voxel’s neighborhood. In particular, the pixel intensity of TDI maps reflects the number of probabilistic streamlines traversing the voxel and the color reflects diffusion streamline orientations similar to those of conventional DTI, thus allowing better WM anatomical localization and characterization (9).

Of note, CSD is based on the estimation, for each voxel, of a Fiber Orientation Distribution (FOD) which describes the number and orientation of the fiber bundles passing through the voxel. The computed FODs can then be used to generate tractograms using different tractography algorithms, allowing the reconstruction of any configuration of fiber tracts. This enables CSD to fully represent WM tract crossing or fanning, producing tract reconstructions that are more faithful to the real anatomy of WM fibers than those obtainable with DTI. The increased precision in reconstructions makes CSD a good candidate for many applications, such as surgical planning, as performed by Küpper et al. (10). In this study, the group compared the reconstructions of the CST of a 6-year-old child with a brain tumor, obtained with three different methodologies (DTI with deterministic tractography, DTI with probabilistic tractography [PT], and CSD-PT) applied on data acquired with 60 diffusion directions and b = 3,000 s/mm^2^; CSD delineated the full tract better than DTI, showing that the tumor included more CST fibers, which was confirmed with electrical stimulation during surgery.

CSD-PT is often applied to the study of motor and language tracts. For example, Liégeois et al. (11, 12) studied which tractography-derived measures best predicted language outcome and presence of dysarthria long term after childhood brain traumatic injury. Northam et al. (13) evaluated the relationship between WM microstructure and speech deficits in very adolescents born very preterm (VPT), with a spectrum of brain injuries. Gordon et al. (14) analyzed reorganization of motor pathways and cortical motor activity in an 11-year-old child who sustained an arterial ischemic stroke in the perinatal period.

In the past few years, CSD has been increasingly used to study WM alterations and functional impairments in children born very preterm (VPT); for example, Murray et al. (15) used this method to analyze the correlation between WM abnormalities and changes in attention ability in VPT children, while Thompson et al. (16) analyzed structural connectivity in VPT children at 7 years of age and found it to correlate with impaired intelligence and movement. Both Thompson et al. (17) and Kelly et al. (18) used CSD to study DTI scalar values and tract volume in the optic radiation of VPT children and to correlate them with visual outcome.

In order to fully exploit the capabilities of the CSD method, specific acquisition parameters are required, including a strong magnetic field (i.e., 3 T or above), a high number of directions (from a minimum of 30 up to 60 and more), and high b-values (optimal value, 3,000 s/mm^2^) (19). All the previously mentioned studies applied parameters in line with these recommendations. This protocol requires a long acquisition time and is often unfeasible in the pediatric clinical setting, in which shorter acquisition sessions are usually preferred with less gradient directions and lower b-values so as to keep the examination as short as possible. This decreases the angular resolution of the acquired images. In addition, pediatric imaging studies are often acquired on 1.5 T scanners. For all these reasons, DTI is often preferred as a processing image analysis method in clinical studies. The aim of this work was to compare the directional maps and reconstruction results of the traditional DTI methodology with those obtainable with TDI maps and CSD and probabilistic tractography (CSD-PT) on data from both myelinated and unmyelinated subjects, acquired with low angular resolution, low b-value diffusion sequences commonly used in clinical studies.

## 2 Materials and Methods

### 2.1 Subjects

All processing and analysis were performed retrospectively on data acquired at the Istituto Giannina Gaslini (Genoa, Italy), from 2011 to 2016. This study was carried out in accordance with the recommendations of the Gaslini Institute review board with written informed consent from all subjects. All subjects gave written informed consent in accordance with the Declaration of Helsinki. The protocol was approved by the local review board. For all subjects, parents signed an informed consent prior to image acquisition.

For this study, we selected the DW-MRI studies of 50 subjects subdivided into three groups: 8 neonates (group I, mean age 11.63 ± 5.3 days), 20 infants (group II, from 1 month to 2 years old, mean age 5 ± 4.32 months), and 22 children and adolescents from 2 to 17 years old (group III, mean age 8.18 ± 4.82 years). All subjects had undergone brain MRI for minor neurological problems, such as headaches, minor trauma, or transient febrile convulsions. All subjects were diagnosed as neurologically and developmentally normal by experienced pediatric neurologists, and all had normal MRI findings.

### 2.2 Image Acquisition

For each subject, a DW-MRI sequence was acquired on a 1.5 T scanner (Philips Intera Achieva version 2.6, Best, the Netherlands). The acquisition sequence was an axial single-shot spin-echo echo-planar sequence commonly used in clinical practice. Acquisition parameters were as follows: 60 axial slices; slice thickness, 2 mm; acquisition matrix, 128 × 128 (in-plane resolution, 1.75 mm × 1.75 mm); TR = 8,129 ms; TE = 80 ms; averages = 1. The signal was acquired along 34 noncollinear directions of space, using a b-value of 1,000 s/mm^2^. One measurement without diffusion weighting (*b* = 0 s/mm^2^) was also performed for each sequence. The average duration of the acquisition sequence was 5 min 34 s. Neonates belonging to group I were fed before MRI examination to achieve spontaneous sleep and were spontaneously breathing during examination. For groups II and III, subjects under 6 years of age or who were uncooperative were sedated during examinations. For all subjects, heart rate and oxygen saturation were monitored by pulse-oximetry throughout the examination. For both sedated and non-sedated subjects, the duration of the examination was the same, in order to keep the sedation period as short as possible.

All brain MRI studies were obtained with axial sections parallel to the bicommissural line, and included additional 3-mm thick T2-weighted images on the three planes of space and a 3D T1 anatomical sequence, with different acquisition parameters based on patient age. These sequences were used to assess the presence of brain lesions or malformations.

### 2.3 Whole-Brain Tractography and Track Density Maps

All diffusion-weighted images were preprocessed with FSL tools (20), correcting for subject movement artifacts and eddy currents. Fiber tracking was performed using the MRtrix package (J-D Tournier, Brain Research Institute, Melbourne, Australia; https://github.com/MRtrix3/mrtrix3) (21). CSD was performed on the preprocessed DWI images in order to estimate the fiber orientation distributions (FODs) in each voxel, using a maximum harmonic degree (*λ_max_*) of 6, which was the maximum value allowed by the data, as described in Tournier et al. (22). From these FODs, streamlines were computed using the iFOD2 algorithm, developed by Tournier et al. (23) and made available by the MRtrix toolbox.

As a first step, we computed a whole-brain tractogram for each subject using the methods described. Each tractogram was composed by 2 million streamlines, with a maximum number of generation trials for the algorithm of 200 million. We tested different combinations of tracking parameters in order to see their effect on the reconstructions, following the work of Tournier et al. (21, 23). Step size was set to the default value of 0.9 mm (about 0.5 times the voxel size, as recommended for the iFOD2 algorithm in Tournier et al. (22)), which was shown to be a good compromise between quality of the results and computational time. As performed in the study by Tournier et al. (21), we tested different values for the cutoff threshold (FOD amplitude value under which the streamlines are terminated) and for the maximum angle between successive tracking steps. In order to find the best combination of values for these parameters, we produced whole-brain tractograms for six representative subjects selected at random from the three groups (two neonates from group I, two infants from group II, and two children from group III), using different combinations of parameters. One expert neuroradiologist examined the results and rated them on a 5-point scale (1, non-diagnostic tracks; 2, poor quality; 3, fair quality; 4, good quality; 5, excellent quality). The combination of parameters which obtained on average the highest scores was selected for the subsequent analyses. The cutoff threshold was set to be 0.2, while the maximum angle between steps was 50°. Table S1 in Supplementary Material shows the results of the rating for the different combinations of values. The maximum and minimum streamline lengths were set, respectively, to 200 mm (about ten times the size of the DWI voxel) and 9 mm (about 5 times the voxel size), as suggested in the toolbox documentation. The generated streamlines were anatomically restrained with a brain mask computed directly from the DWI images with the MRtrix toolbox: streamlines were terminated when they exited the mask. The same brain mask was also used as a seeding mask: generation points for the computed streamlines (“seeds”) were uniformly distributed in the mask, and streamlines were propagated from each seed bidirectionally until termination. The average computation time for this first step (including preprocessing, up to the generation of the whole-brain tractograms) was of 30 min on an iMac desktop workstation (8-core Intel Core i7 @3.5 GHz, 32 GB RAM).

From the whole-brain tractograms, we computed for each subject a gray-scale super resolution TDI map and a directionally encoded color TDI map (DEC-TDI map) (8), with a spatial resolution of 0.5 mm. DEC-TDI maps were colored by assigning red to the right–left direction, green to the anterior–posterior direction, and blue to the inferior–superior direction. In order to improve visualization of the maps, we applied a short-track method to produce short-track DEC-TDI maps (stDEC-TDI maps) as previously reported (9). These modified maps were created from tractograms computed with the same parameters used for the whole-brain tractograms mentioned earlier (FOD amplitude threshold, 0.2; maximum angle between steps, 50°; step size, 0.9 mm), but with a maximum streamline length of 20 mm. In order to maintain a reasonable contrast-to-noise ratio, we generated stDEC-TDI maps from tractograms composed of 20 million streamlines. This number of streamlines was chosen to be an order of magnitude greater than the number of streamlines used for the DEC-TDI maps, as recommended by Calamante et al. (24).

### 2.4 Eigenvector Maps

To provide comparison with the computed TDI maps, we used the MRtrix toolbox to compute the DTI diffusion tensors in each subject. From these diffusion tensors, we computed a color-coded map of the principal eigenvector (EV map) for each subject, in order to compare it with the stDEC-TDI map. EV maps encode, in each voxel, the main direction of diffusion within the voxel, together with its magnitude. The maps were color coded with respect to this main diffusion direction in the same way as the DEC-TDI and stDEC-TDI maps (red for left–right, green for anterior–posterior, blue for inferior–superior) and had the same voxel size as the DWI data.

### 2.5 Anatomical Analysis of Conventional and stTDI Data

Two neuroradiologists, respectively, with 10 and 7 years of experience on pediatric neuroimaging studies, reviewed all MRI studies to perform an image quality assessment. For each subject, they characterized anatomical detail in axial MRI sections from the conventional MRI protocol, EV maps, and short-track TDI parameter maps at 5 canonical anatomical levels of the brain: corpus callosum, internal capsule, rostral midbrain, middle pons, and rostral medulla (similar to Hoch et al. (9)). The TDI maps were then labeled by consensus according to the standard anatomical texts of Duvernoys Atlas of the Human Brain Stem and Cerebellum (25) and of WM atlas mapping (26).

### 2.6 ROI Placement and Reconstruction of WM Tracts

In order to evaluate the performance of CSD, we used a probabilistic tractography method to reconstruct three main WM tracts: the cerebellar-thalamic tracts (CTT), the corticopontocerebellar tracts (CPCT), and the corticospinal tracts (CST). Because of their important roles in voluntary movement control, these tracts are among the main constituents of the WM pathways most commonly investigated by tractography. In particular, the CTT is the main efferent tract from the cerebellum, the CPCT constitutes the main afferent pathway from the cerebral cortex to the cerebellum, and the CST originates from the precentral areas and descends through the centrum semiovale and ipsilateral posterior limb of internal capsule to the brainstem.

The tracts were reconstructed using either a single or a multiple regions of interest (ROI) approach depending on the specific tract, according to WM atlas mapping (26). In each subject, ROIs were drawn separately for the right and left sides on computed DEC-TDI maps, taking advantage of the improved anatomical visualization offered by these maps.

In detail, (i) for the CPCT, we placed a seeding ROI in the middle cerebellar peduncle on the coronal plane and an inclusion ROI in the posterior limb of the internal capsule on the axial plane; (ii) for the CTT, we placed a seeding ROI in the superior cerebellar peduncle on the coronal plane; and (iii) for the CST, we placed the seeding ROI on the posterior limb of the internal capsule, and an additional ROI was placed in the cerebral peduncle, on the right and left sides separately, on the axial plane. The ROIs were drawn by a single operator during a single session, in order to reduce inter-subject variability. All the ROIs were drawn so as to completely include the anatomical structures mentioned earlier, as previously described by Lim et al. (27), thus ensuring that the considered tract would be included in the final reconstruction while diminishing the influence of the ROI choice on the final tract reconstructions (28).

Tractography was performed for each tract with the same algorithm and parameters used to compute the whole-brain tractograms. Each tract was composed of 10,000 streamlines, with a maximum number of generation trials for the algorithm of 1 million. All selected tracts were also reconstructed with a combination of traditional DTI and deterministic tractography (Fiber Assignment by Continuous Tracking (FACT) (29)), in order to validate the CSD reconstruction results and to compare the quality of the tracts obtained with the two methods, using the same ROI placement. The DTI analysis and deterministic tractography were performed using the MRtrix package, which implements the deterministic tractography method on the diffusion-weighted images. Parameters were left at their default values for the toolbox, as defined in the study by Tournier et al. (21). These parameters were found to give the best results for the data, as determined by visual inspection of the results. In particular, the FA threshold (value under which streamlines were terminated) was 0.1 and the maximum angle between steps was 9°. As for CSD tractography, the maximum streamline length was set to 200 mm. Again, each tract was composed of 10,000 streamlines, with a maximum trial number of 1 million. For both methods and all tracts, reconstructions were obtained by seeding streamlines uniformly inside the selected ROIs and propagating them bidirectionally until termination. The same brain mask used for the whole-brain tractograms was used to terminate streamlines when they exited the brain.

### 2.7 Qualitative Analysis of Reconstructed Tracts

Two other neuroradiologists (respectively, with 20 and 3 years of experience on pediatric neuroimaging studies) independently reviewed all tractography results using Trackvis (http://trackvis.org/), a software program that allows interactive visualization of tractography reconstructions. The neuroradiologists performed a track quality assessment by using a 5-point scale (1, non-diagnostic tracks; 2, poor quality; 3, fair quality; 4, good quality; 5, excellent quality). The evaluations were based on the presence of false-positive and false-negative tracts and anatomical accuracy of the reconstructed bundles (30).

### 2.8 Statistical Analysis

For each tract and reconstructing technique, frequencies and percentages of the quality score were calculated across subjects. The average weighted score was computed for each tract and for each evaluator as the average of all scores weighted by their frequency. Chi-square test was used to compare qualitative scores of CSD and DTI tracts. Interobserver agreement was evaluated using the Cohen’s kappa test (31). A K > 0.70 indicated excellent, 0.40–0.70 fair-to-good (moderate), and <0.40 poor agreement (32). Statistical analysis was performed with SPSS Statistics for Mac 21.0 (IBM, Armonk, NY, USA). Results were considered significant at *p* < 0.05.

## 3 Results

### 3.1 EV Maps, Short-Track TDI, and DEC-TDI Maps

Good quality directionally encoded color track density maps were obtained in all patients, as determined by visual inspection. Short-track TDI and DEC-TDI maps better depicted the brain anatomy compared with the conventional images, showing consistent concordance with available anatomical atlases and previous studies (9, 25, 26). The EV maps allowed to visualize the main fiber tracts in older children, while they presented a more blurred appearance for unmyelinated neonates; the distinguishable level of detail was lower in the EV maps than in stDEC-TDI for all subjects.

**Figure 1:**
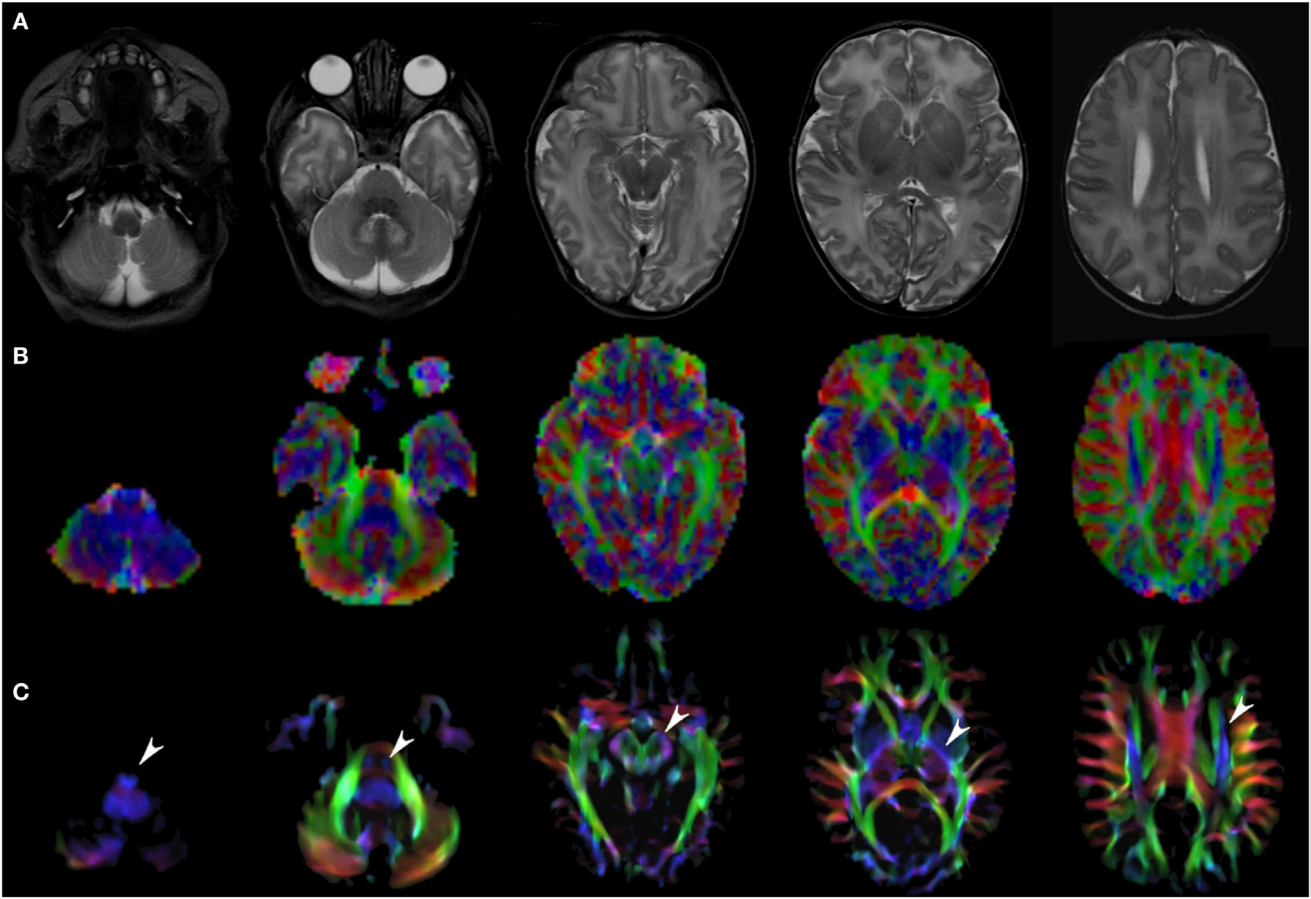
EV maps and short-track TDI maps at 5 canonical brain levels (rostral medulla, middle pons, rostral midbrain, internal capsules, and corpus callosum) in a 6-day neonate. Axial T2-weighted images (**(A)** upper row), corresponding EV maps (**(B)** middle row), and stDEC-TDI images (**(C)** bottom row). Conventional color scheme: blue (inferior–superior), green (anteroposterior), and red (left–right). Note that even in an unmyelinated brain the corticospinal tract is clearly visible from the bulbar level to the centrum semiovale in the stDEC-TDI map (arrowheads), while it is more difficult to distinguish in the EV maps especially at the bulbar level (first column).

**Figure 2:**
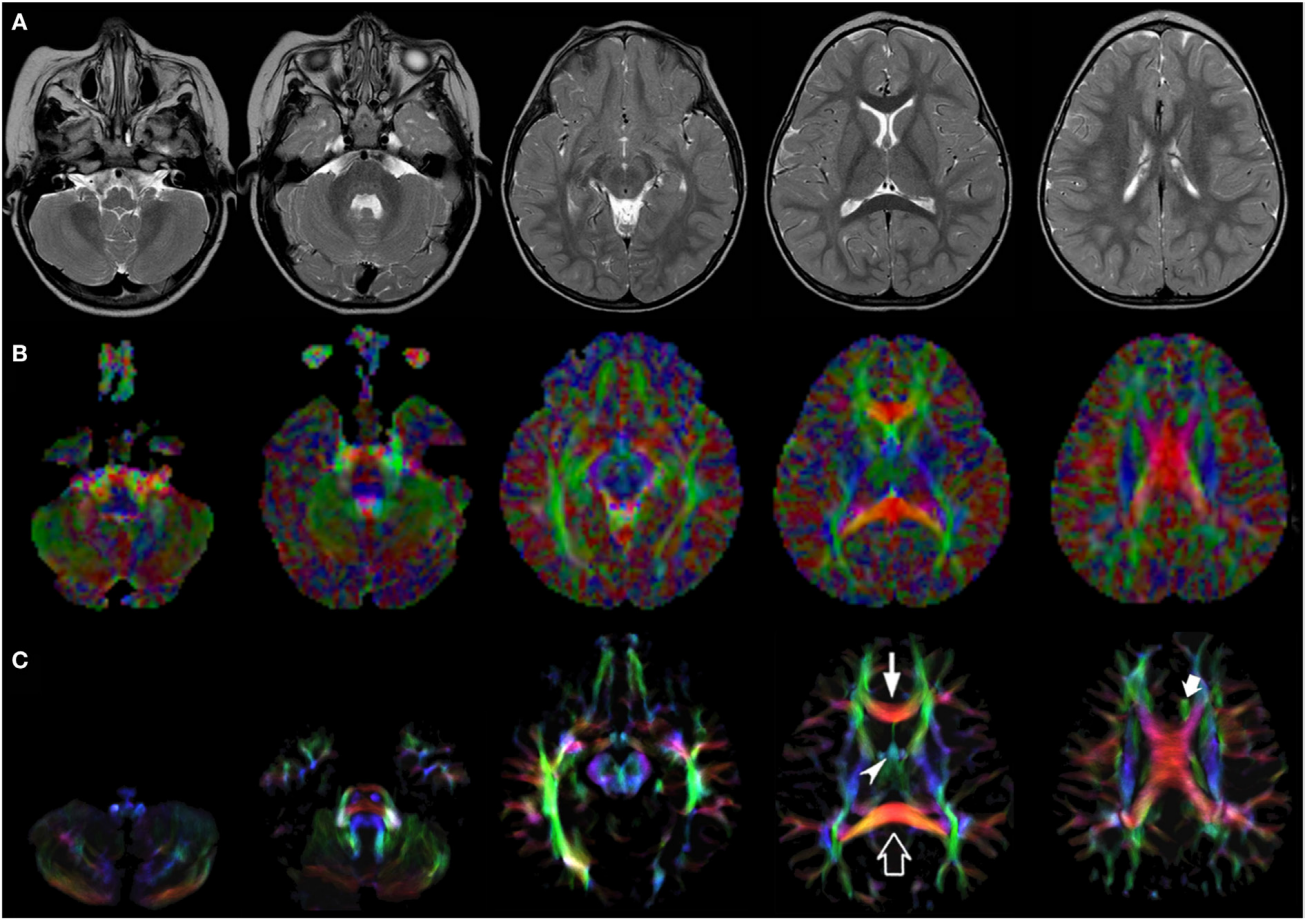
EV maps and short-track TDI maps at 5 canonical brain levels (rostral medulla, middle pons, rostral midbrain, internal capsules, and corpus callosum) in a 16-month-old infant. Axial T2-weighted images (**(A)** upper row), corresponding EV maps (**(B)** middle row), and stDEC-TDI images (**(C)** bottom row). Conventional color scheme: blue (inferior–superior), green (anteroposterior), and red (left–right). The stDEC-TDI images clearly show the genu (thin arrow) and splenium (empty arrow) of the corpus callosum in red. The anterior columns of the fornix (arrowhead) are depicted in light blue. The anterior portion of cingulum (thick arrow) is colored in green. The main WM tracts are distinguishable also in the EV maps, but the quality is lower; the corticospinal tract is almost not distinguishable from non-WM voxels at the bulbar level (first column), while in the stDEC-TDI map it is clearly visible and distinguished from the background.

**Figure 3:**
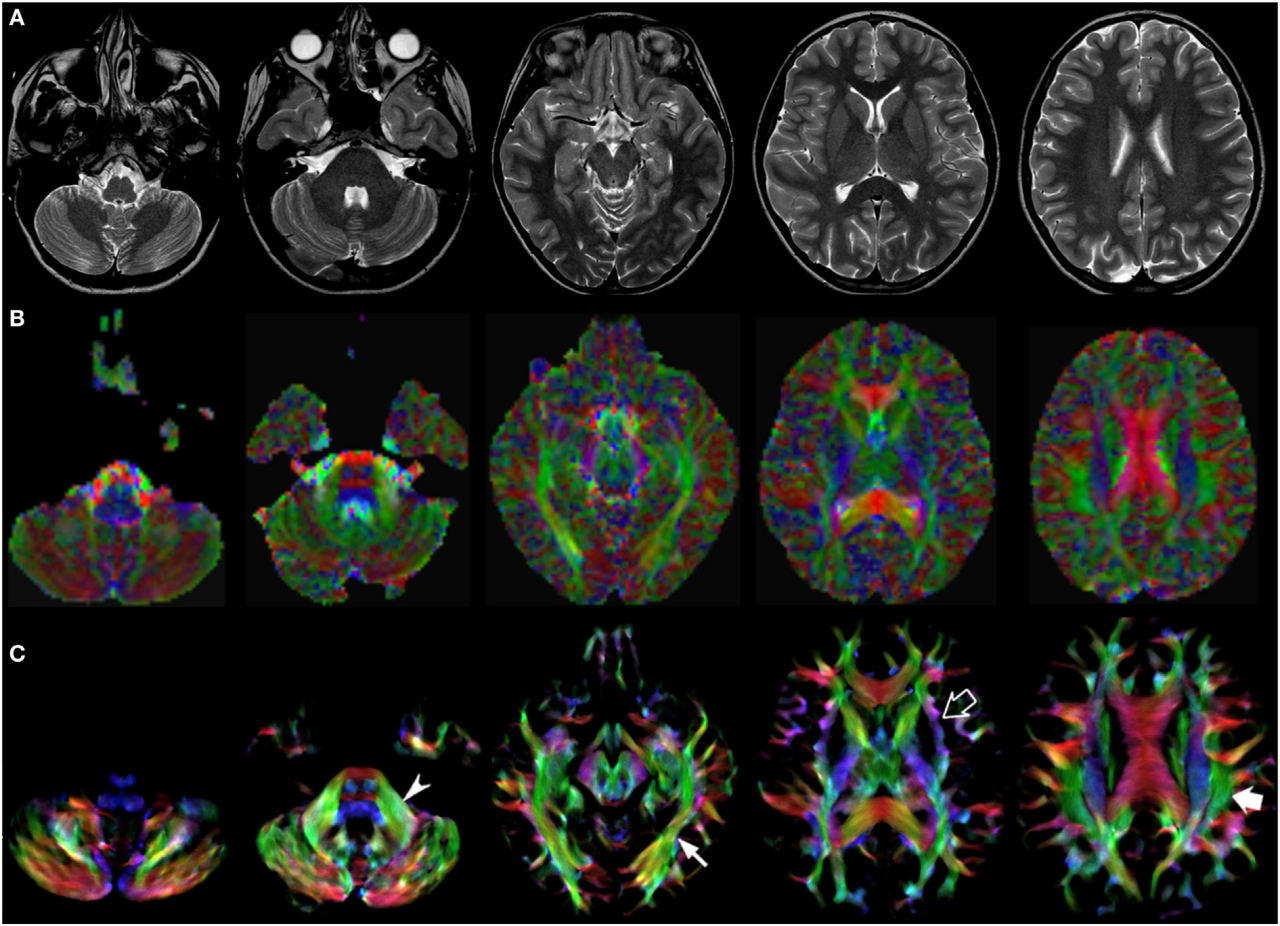
EV maps and short-track TDI maps at 5 canonical brain levels (rostral medulla, middle pons, rostral midbrain, internal capsules, and corpus callosum) in a 10-year-old child. Axial T2-weighted images (**(A)** upper row), corresponding EV maps (**(B)** middle row), and stDEC-TDI images (**(C)** bottom row). Conventional color scheme: blue (inferior–superior), green (anteroposterior), and red (left–right). The stDEC-TDI maps show the middle cerebellar peduncles in green (arrowhead), the inferior longitudinal fasciculi in green (thin arrow), the external capsules in blue–green (empty arrow), and the superior longitudinal fasciculi in green (thick arrow). As in the infant case, the visualization is enhanced in the stDEC-TDI maps and the quality is higher; non-WM voxels show no values, while in the EV maps the WM structures are harder to distinguish from the background voxels.

**Figure 4:**
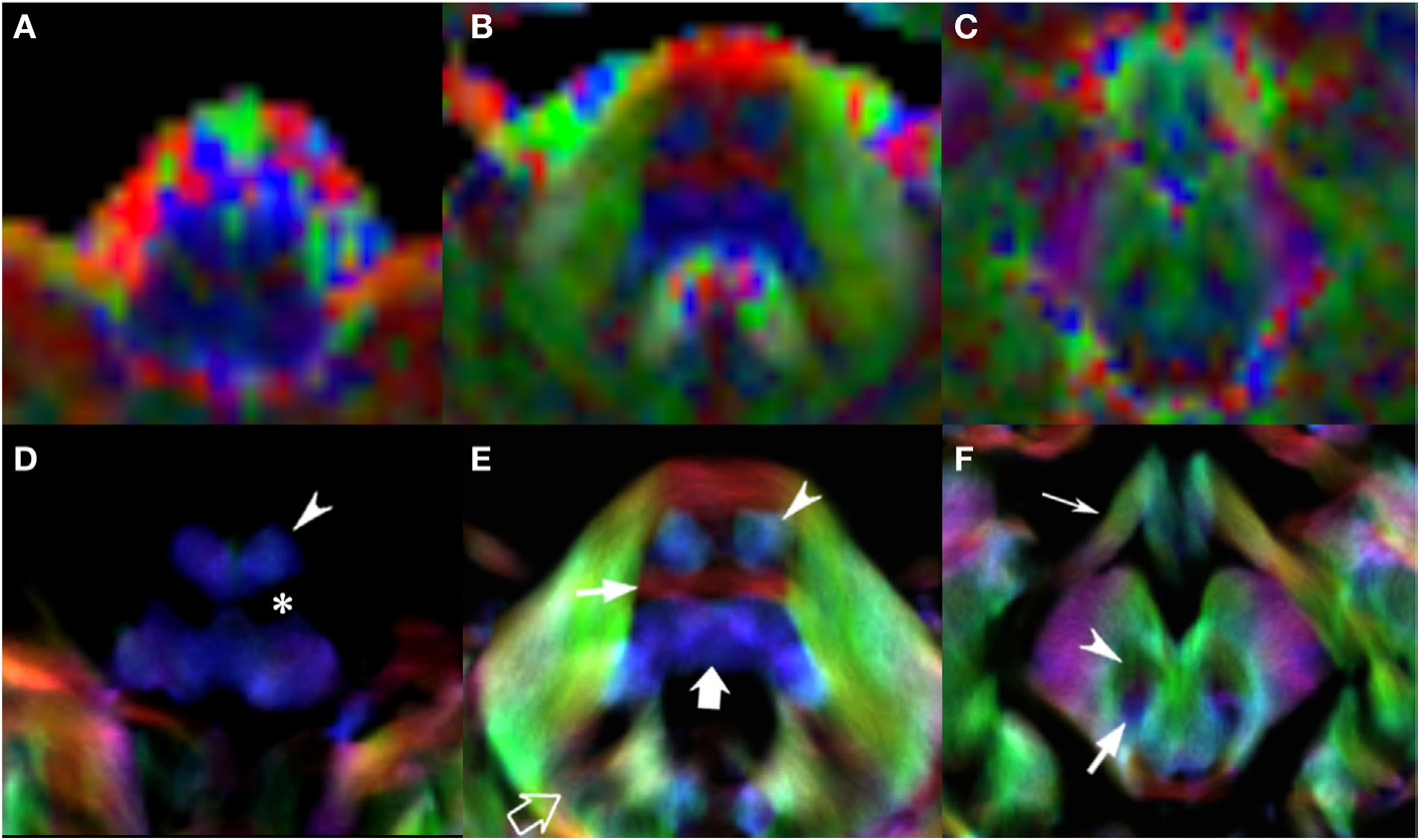
EV maps and short-track TDI maps, magnified view at 3 brainstem levels (rostral medulla, middle pons, and rostral midbrain) in an 8-year-old child. Upper row: EV images. Lower row: stDEC-TDI images. Conventional color scheme: blue (inferior–superior), green (anteroposterior), and red (left–right). First column **(A,D)**: at the bulbar level, the corticospinal tract is on the anterior surface of the pyramids (arrowhead), adjacent to the inferior olivary nucleus (asterisk). In the EV map, non-WM voxels show and “blur” the corticospinal tract, making it more difficult to distinguish. Second column **(B,E)**: at mid-pons level, the pyramidal tract (arrowhead) is clearly separated from the transverse pontocerebellar fibers (thin arrow) and ascending pathways (thick arrow). Note the dentate nuclei (empty arrow), almost not recognizable in the EV map. Third column **(C,F)**: at rostral midbrain, the central tegmental tract (arrow) is located posteriorly to the red nuclei (arrowhead). Note the optic tracts colored in green (thin arrow). The smaller voxel size of the stDEC-TDI maps allows to visualize all the structures correctly even at a zoomed level, while the EV maps at the same level of magnification appear blurred and with a stair-stepped effect.

Figures 1–3 demonstrate axial images of the brain at 5 discrete anatomical levels on conventional imaging, EV maps, and stDEC-TDI maps in one representative case of each age group, respectively. In particular, even in unmyelinated or partially myelinated brains, several WM bundles could be clearly discriminated in the stDEC-TDI maps (Figures 1 and 2), while the same bundles were harder to distinguish in the EV maps, especially at the brainstem levels. In older children, WM tracts and nuclear groups could be visualized with greater anatomical detail in the stDEC-TDI maps than in the EV maps, again particularly in the brainstem (Figure 3). By comparing zoomed versions of the EV and stDEC-TDI maps, the advantage in visualization offered by the stDEC-TDI maps is clear: the reduced voxel size allows to easily visualize and distinguish the WM tracts even when zooming the image, while the EV maps appear blurred and with a stair-step effect. The different anatomical structure is more confused and less easily discriminated in the zoomed EV maps (Figure 4).

### 3.2 CSD and DTI Reconstruction of White Matter Tracts

**Figure 5:**
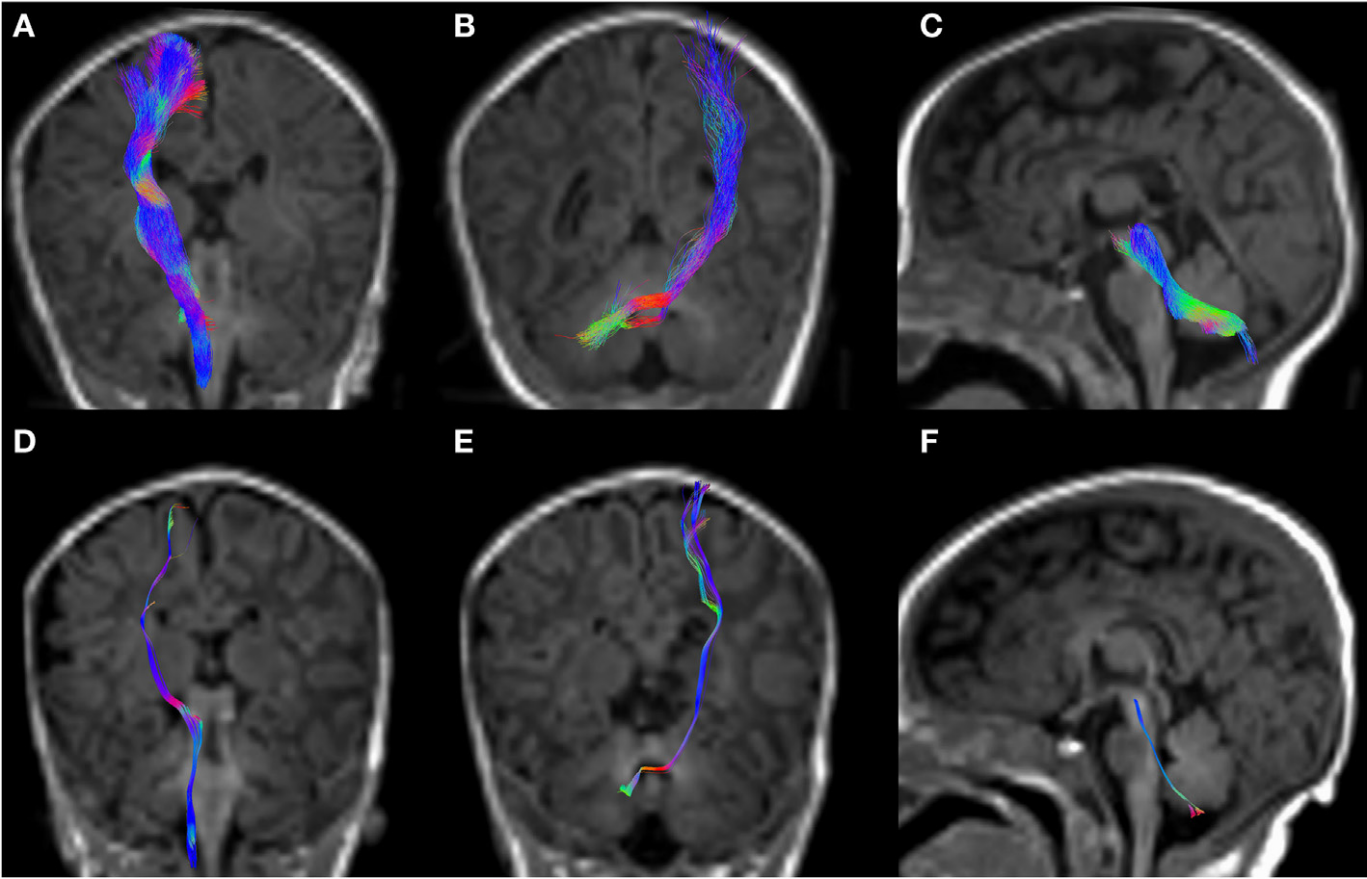
CSD-PT (upper row) and DTI (lower row) reconstructions of the CST (**(A,D)**—first column), CPCT (**(B,E)**—second column), and CTT (**(C,F)**—third column) in a 10-day-old neonate. All tracts are reconstructed with both techniques, but the quality of CSD-PT tracts is superior to that of DTI.

**Figure 6:**
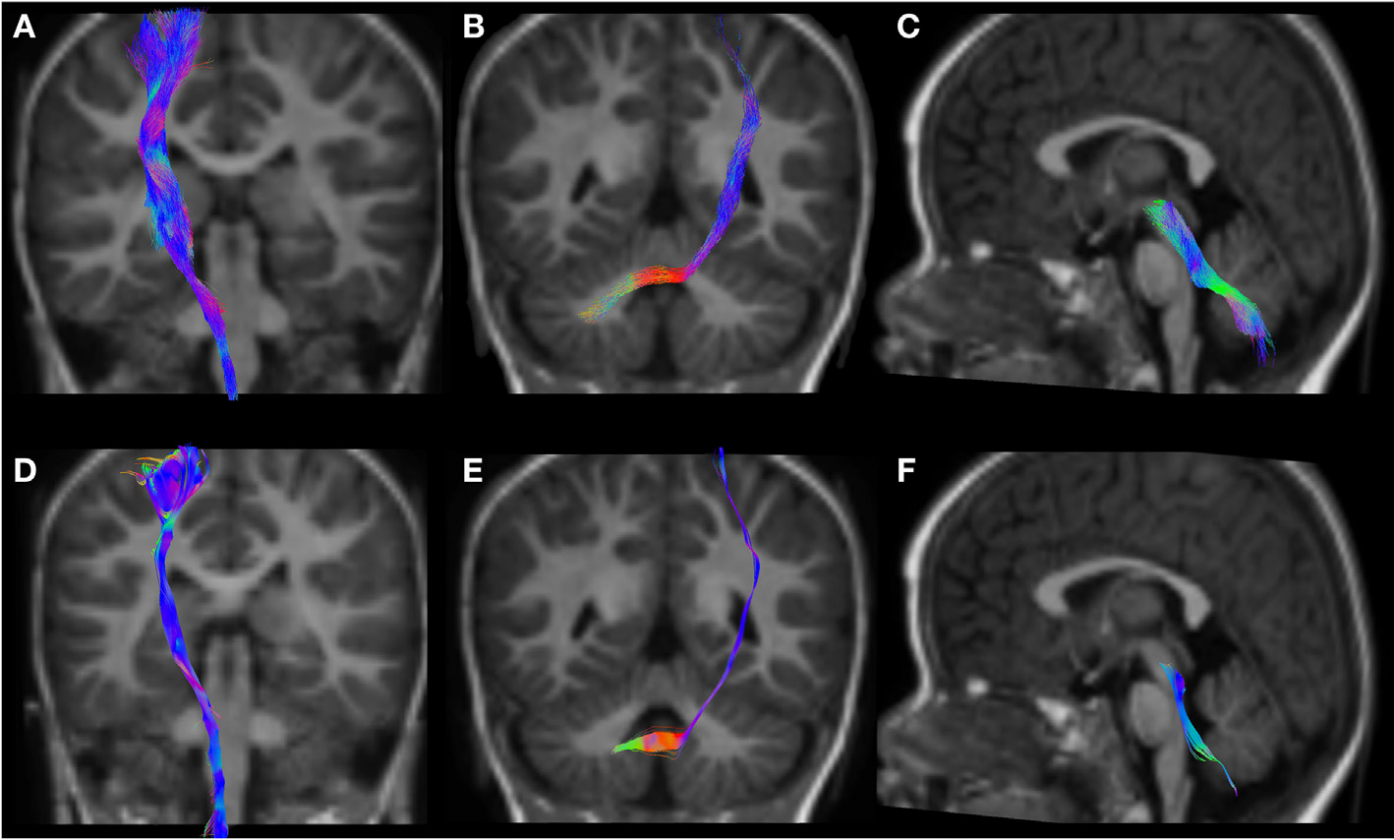
CSD-PT (upper row) and DTI (lower row) reconstructions of the CST (**(A,D)**—first column), CPCT (**(B,E)**—second column), and CTT (**(C,F)**—third column) in a 12-month-old infant. All tracts are reconstructed with both techniques, but the quality of CSD-PT tracts is superior to that of DTI.

**Figure 7:**
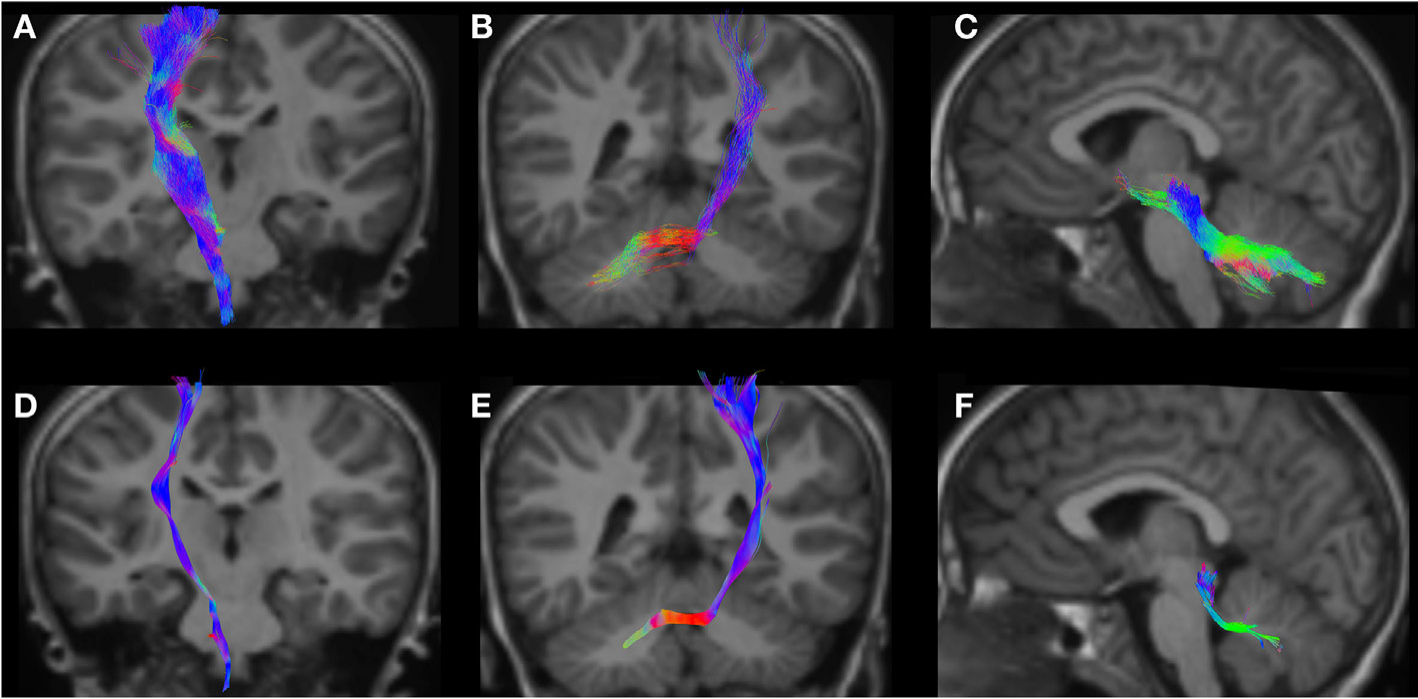
CSD-PT (upper row) and DTI (lower row) reconstructions of the CST (**(A,D)**—first column), CPCT (**(B,E)**—second column), and CTT (**(C,F)**—third column) in a 6-year-old child. All tracts are reconstructed with both techniques, but the quality of CSD-PT tracts is superior to that of DTI.

All tracts were reconstructed with CSD-PT, showing a 100% success rate for CSD-PT fiber tractography reconstruction. DTI fiber reconstruction also showed a 100% success rate, but the reconstructions obtained with CSD-PT received higher quality scores more frequently than the DTI reconstruction. A summary of visual assessment results and inter-reader agreement evaluation are reported in Table 1. Figures 5–7 demonstrate CTT, CPCT, and CST reconstructions using both methods in one representative case of each age group, respectively. While all the considered fiber tracts are reconstructed successfully, the DTI tracts appeared thinner and with a reduced volume with respect to known anatomy, especially in the infant (Figure 6) and neonate (Figure 5). CSD-PT tracts, on the contrary, were fully reconstructed even in unmyelinated neonates, showing the full volume of the WM structures.

**Table 1:** Quality scores with relative frequencies and inter-reader agreements for the DTI and CSD-PT reconstructions of CTT, CPCT, and CST tracts.

Tract	Method	Reader 1			Reader 2			Cohen’s K	*p*-Value
		Score	Frequency	*p*-Value *χ*^2^	Score	Frequency	*p*-Value *χ*^2^		
CTT	DTI	3	18 (36%)	<0.001*	3	15 (30%)	<0.001*	0.60	<0.001*
		4	32 (64%)		4	35 (70%)			
		5	0 (0%)		5	0 (0%)			
	CSD-PT	3	3 (6%)		3	2 (4%)		0.66	<0.001*
		4	28 (56%)		4	27 (54%)			
		5	19 (38%)		5	21 (42%)			
CPCT	DTI	3	25 (50%)	0.008*	3	22 (44%)	0.025*	0.72	<0.001*
		4	25 (50%)		4	28 (56%)			
		5	0 (0%)		5	0 (0%)			
	CSD-PT	3	2 (4%)		3	5 (10%)		0.62	<0.001*
		4	35 (70%)		4	32 (64%)			
		5	13 (26%)		5	13 (26%)			
CST	DTI	3	43 (86%)	0.039*	3	38 (76%)	0.024*	0.66	<0.001*
		4	7 (14%)		4	12 (24%)			
		5	0 (0%)		5	0 (0%)			
	CSD-PT	3	1 (2%)		3	0 (0%)		0.57	<0.001*
		4	20 (40%)		4	25 (50%)			
		5	29 (58%)		5	25 (50%)			

*CTT, cerebellar-thalamic tract; CPCT, corticopontocerebellar tract; CST, corticospinal tract; CSD, constrained spherical deconvolution; DTI, diffusion tensor imaging; p-value χ^2^, p-value for the Chi-square test*.

*The 5-point scale: 1, non-diagnostic; 2, poor quality; 3, fair quality; 4, good quality; 5, excellent quality. Only scores from 3 to 5 are reported because no reconstruction was scored with 1 or 2 points*.

*Bold font and asterisks indicate p-values which were statistically significant*.

The resulting average scores with the relative SDs are reported in Table 2. Average scores for CSD-PT tracts were always higher than DTI scores, with a difference ranging from 0.7 to 1.4 points. The difference between scores for the two methods was always significant (*p* < 0.05 for all tracts and all readers) (Table 1).

**Table 2:** Average scores and SDs for the DTI and CSD-PT reconstructions of CTT, CPCT, and CST tracts.

Tract	Method	Reader 1	Reader 2
CTT	DTI	3.64 ± 0.48	3.70 ± 0.46
	CSD-PT	4.32 ± 0.59	4.38 ± 0.57
CPCT	DTI	3.50 ± 0.51	3.56 ± 0.50
	CSD-PT	4.22 ± 0.51	4.16 ± 0.58
CST	DTI	3.14 ± 0.35	3.24 ± 0.43
	CSD-PT	4.56 ± 0.54	4.50 ± 0.51

**Figure 8:**
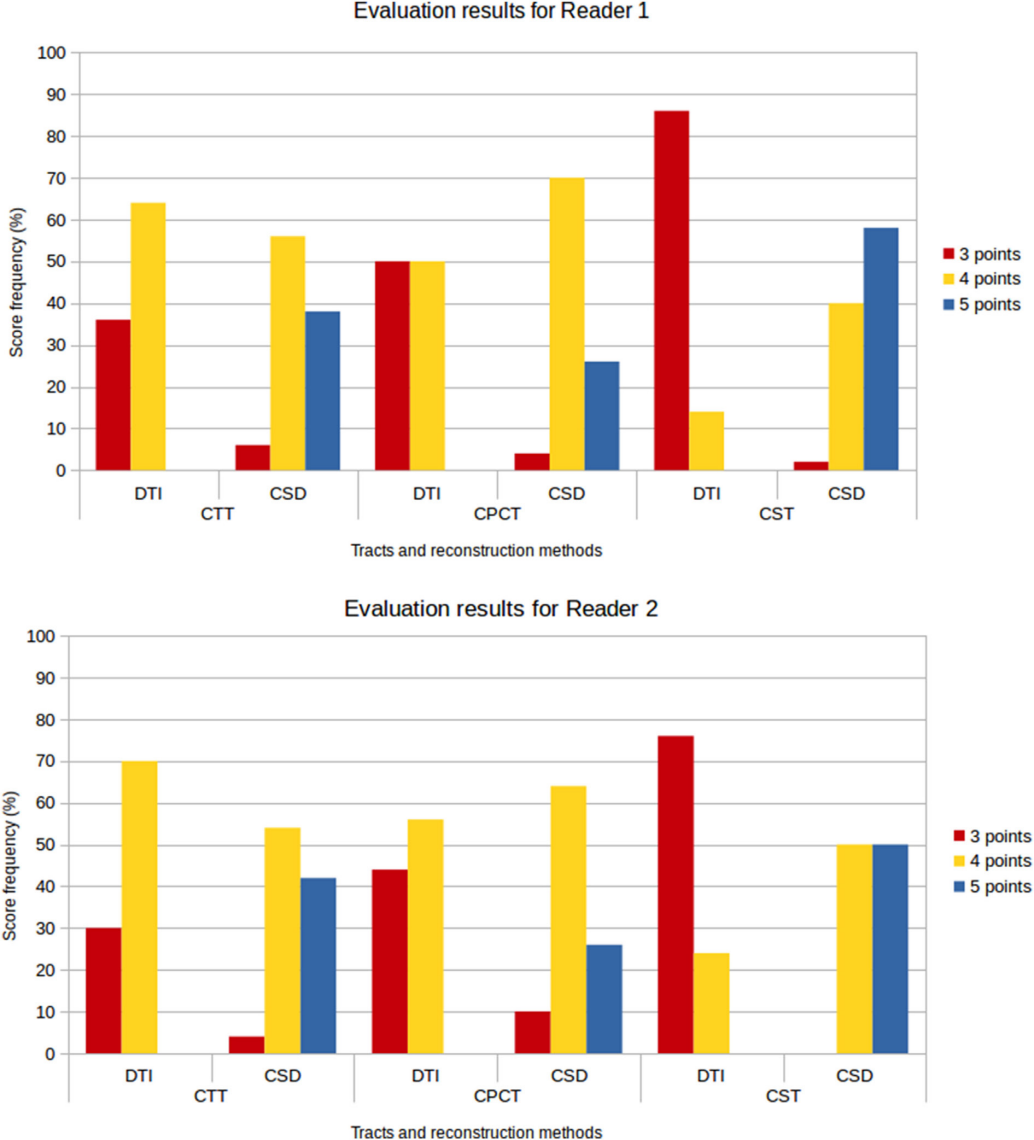
Score frequency histograms for all tracts and reconstruction methods. Top plot: results for Reader 1, bottom plot: results for Reader 2. Only scores from 3 to 5 points are reported in the plots, because no reconstruction was rated 1 or 2 points.

Figure 8 shows the frequency of the scores assigned to all tracts for the different reconstruction methods and for both evaluators. No reconstruction was scored with 1 or 2 points, confirming the 100% success rate for both reconstruction methods; however, lower scores were more frequently awarded to DTI reconstructions, while the frequency of higher scores was higher for CSD-PT reconstructions.

No reconstruction obtained with DTI received the highest score of 5 points, while CSD-PT reconstruction was scored 5 points on average in 40% of the comparisons for CTT, 26% of the comparisons for CPCT, and 54% of the comparisons for CST. For CSD-PT reconstructions of CST, the highest score of 5 points was the most frequently awarded for both readers, while the most frequent score for DTI reconstructions of the same tract was of 3 points. A moderate-to-excellent inter-reader agreement was observed for all qualitative evaluations, with values ranging between 0.6 and 0.7.

## 4 Discussion

In this study, we demonstrated that CSD-PT can be satisfactorily applied to diffusion images acquired with 1.5 T MR scanners and tailored pediatric acquisition protocols, producing good results for clinical applications in neonates and children. In particular, this work shows a 100% success rate for CSD fiber tractography reconstruction of selected WM fiber tracts. Moreover, tracts reconstructed with CSD show better quality with respect to those obtained with a combination of DTI analysis and deterministic tractography, even in neonates with unmyelinated brains.

The TDI maps computed from whole-brain tractograms displayed the WM structure with high resolution, allowing to draw ROIs for the tracts with better precision than T1-weighted anatomical images. These results are in agreement with those of Fiori et al. (33), where cerebellar and corticospinal tracts from children with cerebellar ataxia were compared to those reconstructed from healthy children, in order to analyze microstructural differences. In that study, data were acquired with a 1.5 T scanner in children aged from 4 to 16 years, and ROIs were drawn on short-track TDI maps in order to delineate the tracts more precisely. This practice was also used in Palesi et al. (34, 35), where ROIs drawn on TDI maps were used to reconstruct cerebello-thalamo-cortical pathways in healthy adults.

We found that TDI maps provide WM images of high quality and higher spatial resolution than the original diffusion-weighted data. TDI is performed by mapping reconstructed WM tracks to an image in which the intensity of each voxel reflects the number of tracks passing through that specific position in space. A major advantage of this method is that TDI maps can be computed with a voxel size smaller than the original DWI data, thanks to the interpolation strategy used, thus allowing a better visualization of the results. Directionally encoded color maps can be computed by assigning a color to each spatial direction, and then coloring the map voxels with the correct color depending on the directionality of the fibers in the voxel.

Short-track TDI and DEC-TDI maps allow to better visualize low-intensity fiber tracts and assess the quality of the tractography results. The short maximum length of the reconstructed tracks lowers the TDI contrast in these maps, allowing to better visualize low-intensity structures without oversaturating high-intensity tracts. In order to maintain the contrast-to-noise ratio in the maps, the number of streamlines generated for these maps needs to be about one order of magnitude greater than the number used for normal TDI maps. In this work, the maximum streamline length for the short-track maps was set at 10 times the size of the voxels as previously reported by Calamante et al. (24), so that the length of the streamlines was sufficient to take advantage of neighborhood information added by the super resolution step. The improved visualization of WM tracts was evident in comparison with traditional color-coded EV maps, especially when zooming in on specific WM structures: the smaller voxel allowed to maintain the image quality even at zoomed levels, while the EV maps appeared blurred and confused.

Another advantage of the stTDI and TDI maps over the EV maps is that the TDI maps allow to visualize only the WM tracts, while the EV maps are computed on the whole-brain volume (possibly inside a brain mask). This means that even in voxels where there is no WM (for example, in the ventricles or in the cortex) the EV maps will show diffusion values: this can make the appearance of these maps even more confused, making it more difficult to discriminate between actual WM tracts and other brain structures. This will not happen with TDI and stTDI maps: since they are computed from the tractography results, the maps will have values only in those voxels actually containing reconstructed fibers. Thus, if the tractography results are anatomically correct, the maps will correctly show all WM structures, while voxels containing other tissues will be considered as background. The visualization of WM tracts will thus be easier and less confused by non-WM voxels, as can be appreciated by the figures.

DTI is commonly used to evaluate WM structure in neonates and children, since it is easily applicable on acquisition sequences commonly used in the clinical setting and produces acceptable results. CSD, however, produces reconstructions with better quality and precision, because it can correctly reconstruct configurations with crossing or kissing fibers, which DTI is not able to resolve due to its intrinsic limitations. It is not applied often in the clinical setting, though, because acquisition parameters are often too demanding in terms of scan duration in children and neonates. Among the main factors that influence the choice of processing methodology in a diffusion study, magnet field strength and the parameters of the diffusion-weighted acquisition sequence (mainly the number of gradient directions and the b-value) greatly influence image quality, and thus significantly impact on the yield and applicability of the method in individual cases. Sequences acquired on 1.5 T MR scanners at low b-values (around 1,500 s/mm^2^) are usually processed with DTI analysis to determine diffusion metrics such as fractional anisotropy, using deterministic tractography to extract connectivity measures. Also at 3 T, however, DTI is often applied to neonates and children (36, 37).

In the past few years, several studies have applied CSD to pediatric data acquired with different acquisition parameters. For example, Mormina et al. (38) used CSD-PT to evaluate the structural organization in a 17-year-old patient with cerebellar agenesis, acquiring the images on a 3 T scanner, with 60 gradient directions and a b-value of 1,000 s/mm^2^. Besseling et al. (39) investigated the correlation between functional and structural connectivity in children with rolandic epilepsy compared with healthy controls. Scheck et al. (40) studied the connectivity of the anterior cingulate tract in children with unilateral cerebral palsy, while McGrath et al. (41) analyzed microstructural properties of the inferior fronto-occipital fasciculus and arcuate fasciculus in autistic children. Several groups have applied CSD to study children with ADHD: for example, Silk et al. (42) analyzed WM volume and anomalous asymmetry in DTI scalars of frontostriatal tracts in children from 10 to 19 years old compared to healthy controls. Beare et al. (43) studied altered brain connectivity in ADHD children and adolescents from 9 to 17 years old, using three different methods: CSD-PT, CSD with deterministic tractography, and DTI with FACT tractography. They showed that CSD-PT produced the most stable and robust networks. Dinomais et al. (44) found that long-term motor outcome following neonatal arterial ischemic stroke is closely linked to the extension of the damage to regions of the motor system, by applying CSD-PT to data acquired on a 3 T scanner, with 30 gradient directions and a b-value of 1,000 s/mm^2^.

Other groups have applied these methodologies on pediatric data acquired with a 1.5 T scanner. Lennartsson et al. (45) used CSD-PT to analyze motor and sensory tracts in children around 13 years old, using a sequence with 45 gradient directions and a b-value of 1,000 s/mm^2^. Groeschel et al. (46) also analyzed diffusion parameters along motor tracts in adolescents born very preterm (VPT) and term-born controls, finding microstructural WM alterations in VPT subjects. Fiori et al. (47) found altered connectivity in brain regions associated with speech and language functions in children from 5 to 17 years old suffering from childhood apraxia of speech. Northam et al. (48) also studied language impairment in adolescents born very preterm, finding that damage to interhemispheric WM tracts is associated with language impairment in very preterm subjects. Stefanou et al. (49) compared tensor and non-tensor methods in children from 2 to 17 years old with different motor disorders, confirming the superiority of CSD reconstructions. All these groups scanned children at an age when myelination of the WM fibers is complete. Küpper et al. (50) used CSD-PT to study predictive factors for postoperative grasping ability in 102 patients who underwent hemidisconnection between 10 months and 36 years of age.

Very few studies involving neonates and unmyelinated infants have used CSD-PT. Among these, Pieterman et al. (51) used CSD-PT to study the change in connectivity of specific tracts in neonates in the first weeks of life; the images for this study were acquired on a 3 T scanner, with a DWI sequence better suited to the application of CSD-PT (64 gradient directions and a b-value of 2,500 s/mm^2^). The acquisition time for this sequence was 16 min, and some of the neonates were sedated orally with chloral hydrate during the acquisition session. Recently, Batalle et al. (52) studied brain connectivity and development in 65 neonates under 46-week post-menstrual age, by performing CSD-PT on multi-shell DWI data acquired on a 3 T scanner. Salvan et al. (53) analyzed the microstructure of the arcuate fasciculus in 43 preterm neonates at term-equivalent age, correlating it with linguistic skills at 2 years of age: again, the study was conducted on a 3 T scanner, with high angular resolution data acquired with 64 gradient directions and a b-value of 2,500 s/mm^2^. 33 of the 43 neonates were sedated orally during scans. To our knowledge, no other study was performed with CSD on unmyelinated subjects scanned at 1.5 T with a low b-value and a small number of gradient directions.

Our results corroborate the hypothesis that CSD-PT can be successfully applied using routine clinical MR protocols with low angular resolution and low b-value. Further refinements to the results may be obtained using additional frameworks offered by the MRtrix toolbox. For example, Anatomically Constrained Tractography (ACT) (54) incorporates anatomical priors from a tissue segmentation of a T1 image into the tractography process, in order to improve streamline termination criteria and the biological accuracy of the resulting tractograms. Another interesting method to eliminate reconstruction bias and improve biological plausibility of the tractograms is the Spherical-deconvolution Informed Filtering of Tractograms (SIFT) developed by Smith et al. (55). As discussed in the study by Calamante et al. (56), the application of these two methods also greatly improves the biological meaning of TDI maps and quantitative investigations on these maps. The anatomical information added by the tissue maps used in ACT allows streamlines passing through non-WM regions or having implausible trajectories to be rejected, while SIFT allows to reduce the bias toward major WM tracts in tractography reconstructions.

The ACT framework requires a tissue segmentation map in order to be applied; unfortunately, currently, no automatic algorithm for tissue segmentation has universally been accepted as “gold standard” for neonates and children less than 2 years old. This type of segmentation is difficult to perform on neonatal T1 data, because immature WM myelination produces a low contrast between gray and WM. In the past decade, work has been performed to develop methods for automatic segmentation of the neonatal brain (see for example Devi et al. (57) for a review). For example, Batalle et al. (52) managed to apply ACT in a neonatal group by using neonatal-specific segmentation algorithms. Future work should focus on the improvement of automatic segmentation methods to neonatal studies acquired with different parameters, in order to allow the use of the ACT framework. On the other hand, the application of the SIFT framework does not require tissue segmentations, but uses information from computed FODs to reduce the biases introduced in the tractograms by the streamline propagation technique.

We did not have tissue segmentations for all our subjects, and thus we did not apply the ACT framework to our data. This means that our TDI maps and reconstructions could include some “spurious” streamlines, not precisely corresponding to the biological pathway selected. As noted by Smith et al. (54), the application of ACT to reconstruction of known WM bundles is not essential, although it would make this reconstruction more robust especially in regions on the gray–white matter interface. We also chose not to apply SIFT to our data: thus, the streamline density of the TDI maps and of the reconstructions does not precisely represent the density of the underlying WM. We chose not to use this method, because the aim of this work was to qualitatively compare the results from CSD-PT and DTI on low angular resolution data, without any quantitative analysis of TDI maps or tract reconstructions.

Another interesting fiber visualization method which can be applied on FODs computed with CSD is Line Integral Convolution with multidirectional Anisotropic Glyph samples (A-Glyph LIC), proposed by Höller et al. (58). This method applies a multiple kernel LIC algorithm on cylindrical glyphs computed from FODs obtained with CSD. The color-coded maps produced with this algorithm allow to better visualize local diffusion properties with respect to the FA maps obtained with DTI, providing more information about local fiber architecture with respect also to tractography-based approaches. Moreover, this algorithm is fully automated and does not require parameter tuning nor user interaction, making it a valuable tool for visualizing WM in different pathologies. The authors tested this method on four patient datasets, three of which from young patients (between 6.5 and 15 years old). It would be interesting to test the A-Glyph LIC also on neonatal data, to determine its applicability and the improvements with respect to DTI-derived FA maps in such cases.

The CSD-PT technique allows to reconstruct WM tracts more completely than DTI, because it is able to resolve the problem of crossing fiber tracts, which DTI cannot address satisfactorily. The algorithms used in this work were shown to be robust to noise in the data and to produce results consistent with known anatomy. Tournier et al. (7) showed that the FODs estimated with the CSD technique maintain good angular resolution and noise sensitivity even at high noise levels. The iFOD2 algorithm described in the study by Tournier et al. (23) is capable of tracking different conformations of fiber tracts with high accuracy, highly reducing the incidence of biologically unrealistic tracts in the final tractogram. Compared with several reconstruction techniques, CSD-PT has been shown to produce the most complete and accurate fiber reconstructions ((43); see Wilkins et al. (59) for a comparison of several reconstruction techniques, including CSD-PT). As described in the study by Tournier et al. (21), deterministic tracking algorithms usually produce cleaner and less noisy results than PT, but these methods often cause some portions of the considered tracts to be excluded from the reconstructions, especially in case of major fiber tracts crossing or fanning out. The reconstructions obtained in this work confirm this finding (Figures 5–7).

Pediatric DW imaging is challenging, especially in neonates, due to several issues as evidenced in the study by Yepes-Calderon et al. (60). The overall image quality is lower than that of an adult image acquired with the same sequence, because of the lower myelin content of the neonatal brain and the similar water content of WM and GM. The average diffusivity is higher in the neonatal brain than in the adult brain, as described by Hüppi and Dubois (61). Moreover, it is difficult to keep a non-sedated neonate immobile during the acquisition sequence. Shorter DWI acquisition sessions with few gradient directions and lower b-values are usually applied so as to keep the examination as short as possible. As shown in Tournier et al. (19), using a low b-value and a small number of gradient directions, the computed FODs will have a less sharp profile than those obtainable with higher b-values and more gradient directions. The quality of the results will obviously be lower than that obtainable with a better acquisition sequence.

When using CSD-PT to obtain WM reconstruction in the clinical setting, with an acquisition sequence similar to the one used in this work, it will be necessary to accurately examine the results to assess their biological accuracy and plausibility, especially when imaging unmyelinated neonates. The quality of the results will also benefit from using the most uniform spatial distribution of the gradient directions possible: any imperfection in gradient spatial distribution will result in an increase in noise levels of the DW data, as discussed in the study by Tournier et al. (19). In the cited work, the authors suggest that the addition of a few additional gradient directions can be sufficient to overcome this problem.

In conclusion, the improved quality of our reconstruction results shows that methods for advanced diffusion analysis—and in particular CSD—can be applied also to pediatric, including neonatal, brain MR studies performed on low-field scanners and using suboptimal acquisition parameters. A wider application of these methodologies could help clinicians to better understand microstructural WM involvement in several pathologic conditions affecting the pediatric brain.

## Ethics Statement

This study was carried out in accordance with the recommendations of the Gaslini Institute review board with written informed consent from all subjects. All subjects gave written informed consent in accordance with the Declaration of Helsinki. The protocol was approved by the local review board.

## Author Contributions

BT, DT, and MS collected the data. BT ran the analyses. DT and MS validated, as experts, the results. BT, DT, MS, GA, AC, GM, AR, and MF designed the study and wrote the manuscript.

## Conflict of Interest Statement

The authors declare that the research was conducted in the absence of any commercial or financial relationships that could be construed as a potential conflict of interest.

## References

[1] Schaefer PW Grant PE Gonzalez RG. Diffusion-weighted MR imaging of the brain. Radiology (2000) 217(2):331–45. 10.1148/radiology.217.2.r00nv24331 11058626

[2] Moritani T Ekholm S Westesson P-LA Diffusion-Weighted MR Imaging of the Brain. Berlin, Heidelberg: Springer Science & Business Media (2009).

[3] Basser PJ Mattiello J LeBihan D MR diffusion tensor spectroscopy and imaging. Biophys J (1994) 66(1):259 10.1016/S0006-3495(94)80775-1 8130344 PMC1275686

[4] Abhinav K Yeh FC Pathak S Suski V Lacomis D Friedlander RM Advanced diffusion MRI fiber tracking in neurosurgical and neurodegenerative disorders and neuroanatomical studies: a review. Biochim Biophys (2014) 1842(11):2286–97. 10.1016/j.bbadis.2014.08.002 25127851

[5] Daducci A Canales-Rodríguez EJ Descoteaux M Garyfallidis E Gur Y Lin Y-C Quantitative comparison of reconstruction methods for intra-voxel fiber recovery from diffusion MRI. IEEE Trans Med Imaging (2014) 33(2):384–99. 10.1109/TMI.2013.2285500 24132007

[6] Tournier J-D Calamante F Gadian DG Connelly A. Direct estimation of the fiber orientation density function from diffusion-weighted MRI data using spherical deconvolution. Neuroimage (2004) 23(3):1176–85. 10.1016/j.neuroimage.2004.07.037 15528117

[7] Tournier J-D Calamante F Connelly A. Robust determination of the fibre orientation distribution in diffusion MRI: non-negativity constrained super-resolved spherical deconvolution. Neuroimage (2007) 35(4):1459–72. 10.1016/j.neuroimage.2007.02.016 17379540

[8] Calamante F Tournier J-D Jackson GD Connelly A Track-density imaging (TDI): super-resolution white matter imaging using whole-brain track-density mapping. Neuroimage (2010) 53(4):1233–43. 10.1016/j.neuroimage.2010.07.024 20643215

[9] Hoch M Chung S Ben-Eliezer N Bruno M Fatterpekar G Shepherd T. New clinically feasible 3T MRI protocol to discriminate internal brain stem anatomy. AJNR Am J Neuroradiol (2016) 37(6):1058–65. 10.3174/ajnr.A4685 26869471 PMC4907846

[10] Küpper H Groeschel S Alber M Klose U Schuhmann MU Wilke M Comparison of different tractography algorithms and validation by intraoperative stimulation in a child with a brain tumor. Neuropediatrics (2015) 46(1):72–5. 10.1055/s-0034-1395346 25535700

[11] Liégeois FJ Mahony K Connelly A Pigdon L Tournier JD Morgan AT Pediatric traumatic brain injury: language outcomes and their relationship to the arcuate fasciculus. Brain Lang (2013) 127(3):388–98. 10.1016/j.bandl.2013.05.003 23756046 PMC3988975

[12] Liégeois F Tournier JD Pigdon L Connelly A Morgan AT Corticobulbar tract changes as predictors of dysarthria in childhood brain injury. Neurology (2013) 80(10):926–32. 10.1212/WNL.0b013e3182840c6d 23390172

[13] Northam GB Liégeois F Chong WK Baker K Tournier JD Wyatt JS Speech and oromotor outcome in adolescents born preterm: relationship to motor tract integrity. J Pediatr (2012) 160(3):402–9. 10.1016/j.jpeds.2011.08.055 22000302 PMC3657185

[14] Gordon AL Wood A Tournier JD Hunt RW. Corticospinal tract integrity and motor function following neonatal stroke: a case study. BMC Neurol (2012) 12(1):53. 10.1186/1471-2377-12-53 22776078 PMC3464897

[15] Murray AL Thompson DK Pascoe L Leemans A Inder TE Doyle LW White matter abnormalities and impaired attention abilities in children born very preterm. Neuroimage (2016) 124:75–84. 10.1016/j.neuroimage.2015.08.044 26318524 PMC4791057

[16] Thompson DK Chen J Beare R Adamson CL Ellis R Ahmadzai ZM Structural connectivity relates to perinatal factors and functional impairment at 7 years in children born very preterm. Neuroimage (2016) 134:328–37. 10.1016/j.neuroimage.2016.03.070 27046108 PMC4912891

[17] Thompson DK Thai D Kelly CE Leemans A Tournier JD Kean MJ Alterations in the optic radiations of very preterm children – perinatal predictors and relationships with visual outcomes. Neuroimage Clin (2014) 4:145–53. 10.1016/j.nicl.2013.11.007 24371797 PMC3871291

[18] Kelly CE Cheong JLY Molloy C Anderson PJ Lee KJ Burnett AC Neural correlates of impaired vision in adolescents born extremely preterm and/or extremely low birthweight. PLoS One (2014) 9(3):e93188 10.1371/journal.pone.0093188 24663006 PMC3964000

[19] Tournier J-D Calamante F Connelly A. Determination of the appropriate b value and number of gradient directions for high-angular-resolution diffusion-weighted imaging. NMR Biomed (2013) 26(12):1775–86. 10.1002/nbm.3017 24038308

[20] Jenkinson M Beckmann CF Behrens TEJ Woolrich MW Smith SM. FSL. Neuroimage (2012) 62(2):782–90. 10.1016/j.neuroimage.2011.09.015 21979382

[21] Tournier J-D Calamante F Connelly A MRtrix: diffusion tractography in crossing fiber regions. Int J Imaging Syst Technol (2012) 22(1):53–66. 10.1002/ima.22005

[22] Tournier JD Calamante F Connelly A How many diffusion gradient directions are required for HARDI? Proceedings of the International Society for Magnetic Resonance in Medicine (2009). Available from: http://cds.ismrm.org/protected/09MProceedings/files/00358.pdf

[23] Tournier J-D Calamante F Connelly A Improved probabilistic streamlines tractography by 2nd order integration over fibre orientation distributions. Proceedings of the International Society for Magnetic Resonance in Medicine (2010). Available from: http://cds.ismrm.org/protected/10MProceedings/files/1670_4298.pdf

[24] Calamante F Tournier JD Kurniawan ND Yang Z Gyengesi E Galloway GJ Super-resolution track-density imaging studies of mouse brain: comparison to histology. Neuroimage (2012) 59(1):286–96. 10.1016/j.neuroimage.2011.07.014 21777683

[25] Naidich T Duvernoy H Delman B Sorensen A Kollias S Haacke E Duvernoy’s Atlas of the Human Brain Stem and Cerebellum. (Vol. 30). Vienna: Springer Vienna (2009). 10.1007/978-3-211-73971-6

[26] Catani M de Schotten MT. A diffusion tensor imaging tractography atlas for virtual in vivo dissections. Cortex (2008) 44(8):1105–32. 10.1016/j.cortex.2008.05.004 18619589

[27] Lim JC Phal PM Desmond PM Nichols AD Kokkinos C Danesh-Meyer HV Probabilistic MRI tractography of the optic radiation using constrained spherical deconvolution: a feasibility study. PLoS One (2015) 10(3):e0118948. 10.1371/journal.pone.0118948 25742640 PMC4351098

[28] Huang H Zhang J van Zijl P Mori S. Analysis of noise effects on DTI-based tractography using the brute-force and multi-ROI approach. Magn Reson Med (2004) 52(3):559–65. 10.1002/mrm.20147 15334575

[29] Mori S Crain BJ Chacko VP Van Zijl PCM Three-dimensional tracking of axonal projections in the brain by magnetic resonance imaging. Ann Neurol (1999) 45(2):265–9. 10.1002/1531-8249(199902)45:2<265:AID-ANA21>3.0.CO;2-3 9989633

[30] Beddy P Rangarajan RD Kataoka M Moyle P Graves MJ Sala E. T1-weighted fat-suppressed imaging of the pelvis with a dual-echo Dixon technique: initial clinical experience. Radiology (2011) 258(2):583–9. 10.1148/radiol.10100912 21079201

[31] Cohen J A coefficient of agreement for nominal scales. Educ Psychol Meas (1960) 20(1):37–46. 10.1177/001316446002000104

[32] Fleiss J Levin B Cho Paik M Statistical Methods for Rates and Proportions. Hoboken, NJ: John Wiley & Sons (2003). 800 p.

[33] Fiori S Poretti A Pannek K Del Punta R Pasquariello R Tosetti M Diffusion tractography biomarkers of pediatric cerebellar hypoplasia/atrophy: preliminary results using constrained spherical deconvolution. AJNR Am J Neuroradiol (2016) 37(5):917–23. 10.3174/ajnr.A4607 26659337 PMC7960301

[34] Palesi F Tournier JD Calamante F Muhlert N Castellazzi G Chard D Contralateral cerebello-thalamo-cortical pathways with prominent involvement of associative areas in humans in vivo. Brain Struct Funct (2015) 220(6):3369–84. 10.1007/s00429-014-0861-2 25134682 PMC4575696

[35] Palesi F Tournier J-D Calamante F Muhlert N Castellazzi G Chard D Reconstructing contralateral fiber tracts: methodological aspects of cerebello-thalamo-cortical pathway reconstruction. Funct Neurol (2016) 31(4):229–38. 10.11138/FNeur/2016.31.4.229 28072383 PMC5231885

[36] Brown CJ Miller SP Booth BG Andrews S Chau V Poskitt KJ Structural network analysis of brain development in young preterm neonates. Neuroimage (2014) 101:667–80. 10.1016/j.neuroimage.2014.07.030 25076107

[37] Van Den Heuvel MP Kersbergen KJ De Reus MA Keunen K Kahn RS Groenendaal F The neonatal connectome during preterm brain development. Cereb Cortex (2015) 25(9):3000–13. 10.1093/cercor/bhu095 24833018 PMC4537441

[38] Mormina E Briguglio M Morabito R Arrigo A Marino S Di Rosa G A rare case of cerebellar agenesis: a probabilistic constrained spherical deconvolution tractographic study. Brain Imaging Behav (2016) 10(1):158–67. 10.1007/s11682-015-9377-5 25832852

[39] Besseling RMH Jansen JFA Overvliet GM van der Kruijs SJM Ebus SCM deLouw AJA Delayed convergence between brain network structure and function in rolandic epilepsy. Front Hum Neurosci (2014) 8:704. 10.3389/fnhum.2014.00704 25249968 PMC4158874

[40] Scheck SM Pannek K Raffelt DA Fiori S Boyd RN Rose SE. Structural connectivity of the anterior cingulate in children with unilateral cerebral palsy due to white matter lesions. Neuroimage Clin (2015) 9:498–505. 10.1016/j.nicl.2015.09.014 26640762 PMC4610959

[41] McGrath J Johnson K O’Hanlon E Garavan H Gallagher L Leemans A. White matter and visuospatial processing in autism: a constrained spherical deconvolution tractography study. Autism Res (2013) 6(5):307–19. 10.1002/aur.1290 23509018

[42] Silk TJ Vilgis V Adamson C Chen J Smit L Vance A Abnormal asymmetry in frontostriatal white matter in children with attention deficit hyperactivity disorder. Brain Imaging Behav (2016) 10(4):1080–9. 10.1007/s11682-015-9470-9 26525887

[43] Beare R Adamson C Bellgrove MA Vilgis V Vance A Seal ML Altered structural connectivity in ADHD: a network based analysis. Brain Imaging Behav (2017) 11(3):846–58. 10.1007/s11682-016-9559-9 27289356

[44] Dinomais M Hertz-Pannier L Groeschel S Chabrier S Delion M Husson B Long term motor function after neonatal stroke: lesion localization above all. Hum Brain Mapp (2015) 36(12):4793–807. 10.1002/hbm.22950 26512551 PMC6869692

[45] Lennartsson F Holmström L Eliasson AC Flodmark O Forssberg H Tournier JD Advanced fiber tracking in early acquired brain injury causing cerebral palsy. AJNR Am J Neuroradiol (2015) 36(1):181–7. 10.3174/ajnr.A4072 25169928 PMC7965937

[46] Groeschel S Tournier JD Northam GB Baldeweg T Wyatt J Vollmer B Identification and interpretation of microstructural abnormalities in motor pathways in adolescents born preterm. Neuroimage (2014) 87:209–19. 10.1016/j.neuroimage.2013.10.034 24185027

[47] Fiori S Guzzetta A Mitra J Pannek K Pasquariello R Cipriani P Neuroanatomical correlates of childhood apraxia of speech: a connectomic approach. Neuroimage Clin (2016) 12:894–901. 10.1016/j.nicl.2016.11.003 27882295 PMC5114583

[48] Northam GB Liégeois F Tournier JD Croft LJ Johns PN Chong WK Interhemispheric temporal lobe connectivity predicts language impairment in adolescents born preterm. Brain (2012) 135(12):3781–98. 10.1093/brain/aws276 23144265 PMC4031625

[49] Stefanou MI Lumsden DE Ashmore J Ashkan K Lin JP Charles-Edwards G. Tensor and non-tensor tractography for the assessment of the corticospinal tract of children with motor disorders: a comparative study. Neuroradiology (2016) 58(10):1005–16. 10.1007/s00234-016-1721-y 27447871

[50] Küpper H Kudernatsch M Pieper T Groeschel S Tournier JD Raffelt D Predicting hand function after hemidisconnection. Brain (2016) 139(9):2456–68. 10.1093/brain/aww170 27383529

[51] Pieterman K Batalle D Dudink J Tournier J-D Hughes EJ Barnett M Cerebello-cerebral connectivity in the developing brain. Brain Struct Funct (2017) 222:1625–34. 10.1007/s00429-016-1296-8 27573027 PMC5406415

[52] Batalle D Hughes EJ Zhang H Tournier J-D Tusor N Aljabar P Early development of structural networks and the impact of prematurity on brain connectivity. Neuroimage (2017) 149:379–92. 10.1016/j.neuroimage.2017.01.065 28153637 PMC5387181

[53] Salvan P Tournier JD Batalle D Falconer S Chew A Kennea N Language ability in preterm children is associated with arcuate fasciculi microstructure at term. Hum Brain Mapp (2017) 38(8):3836–47. 10.1002/hbm.23632 28470961 PMC5518442

[54] Smith RE Tournier J-D Calamante F Connelly A. Anatomically-constrained tractography: improved diffusion MRI streamlines tractography through effective use of anatomical information. Neuroimage (2012) 62(3):1924–38. 10.1016/j.neuroimage.2012.06.005 22705374

[55] Smith RE Tournier J-D Calamante F Connelly A. SIFT: spherical-deconvolution informed filtering of tractograms. Neuroimage (2013) 67:298–312. 10.1016/j.neuroimage.2012.11.049 23238430

[56] Calamante F Smith RE Tournier J-D Raffelt D Connelly A. Quantification of voxel-wise total fibre density: investigating the problems associated with track-count mapping. Neuroimage (2015) 117:284–93. 10.1016/j.neuroimage.2015.05.070 26037054

[57] Devi CN Chandrasekharan A Sundararaman V Alex ZC Neonatal brain MRI segmentation: a review. Comput Biol Med (2015) 64:163–78. 10.1016/j.nicl.2016.11.003 26189155

[58] Höller M Ehricke HH Synofzik M Klose U Groeschel S Clinical application of fiber visualization with LIC maps using multidirectional anisotropic glyph samples (A-Glyph LIC). Clin Neuroradiol (2015):1–11. 10.1007/s00062-015-0486-8 PMC5577151 26614208

[59] Wilkins B Lee N Gajawelli N Law M Leporé N. Fiber estimation and tractography in diffusion MRI: development of simulated brain images and comparison of multi-fiber analysis methods at clinical b-values. Neuroimage (2015) 109:341–56. 10.1016/j.neuroimage.2014.12.060 25555998 PMC4600612

[60] Yepes-Calderon F Lao Y Fillard P Nelson MD Panigrahy A Lepore N Tractography in the clinics: implementing a pipeline to characterize early brain development. Neuroimage Clin (2017) 14:629–40. 10.1016/j.nicl.2016.12.029 28348954 PMC5357703

[61] Hüppi PS Dubois J. Diffusion tensor imaging of brain development. Semin Fetal Neonatal Med (2006) 11(6):489–97. 10.1016/j.siny.2006.07.006 16962837

