# Supplementary material for: Improvement in White Matter Tract Reconstruction with Constrained Spherical Deconvolution and Track Density Mapping in Low Angular Resolution Data: A Pediatric Study and Literature Review
Source: Front Pediatr. 2017 Aug 30;5:182. doi: 10.3389/fped.2017.00182 (PMC5582070; doi:10.3389/fped.2017.00182)
Supplement: Supplementary file 1 [file table_1.pdf]

# *Supplementary Material*: Improvement of white matter tract reconstruction with constrained spherical deconvolution and track-density mapping in low angular resolution data: a pediatric study and literature review

**Benedetta Toselli***, **Domenico Tortora, Mariasavina Severino, Gabriele Arnulfo, Andrea Canessa, Giovanni Morana, Andrea Rossi and Marco Massimo Fato**

*Correspondence:
Benedetta Toselli
viale Causa 13
16145 Genova (IT)
+39 0103532789
benedetta.toselli@dibris.unige.it

## 1 SUPPLEMENTARY TABLES AND FIGURES

**Supplementary Table 1:** Average scores for whole-brain CSD-PT tractograms obtained with different values of FOD amplitude threshold (0.1, 0.2, 0.3 and 0.4) and maximum angle between tracking steps (10°, 30°, 50°, 70°, 90°). For visualization purposes, a dash ("-") marks cells where the score frequency was 0.0%.

| Max angle | FOD amplitude | Score frequency 1 | 2 | 3 | 4 | 5 |
|---|---|---|---|---|---|---|
| 10° | 0.1 | 100% | - | - | - | - |
| | 0.2 | 100% | - | - | - | - |
| | 0.3 | 100% | - | - | - | - |
| | 0.4 | 100% | - | - | - | - |
| 30° | 0.1 | 33.3% | 66.7% | - | - | - |
| | 0.2 | - | - | 100% | - | - |
| | 0.3 | 16.7% | 83.3% | - | - | - |
| | 0.4 | 100% | - | - | - | - |
| 50° | 0.1 | - | - | 66.7% | 33.3% | - |
| | 0.2 | - | - | - | 66.7% | 33.3% |
| | 0.3 | - | 16.7% | 83.3% | - | - |
| | 0.4 | 100% | - | - | - | - |
| 70° | 0.1 | - | - | 100% | - | - |
| | 0.2 | - | - | - | 100% | - |
| | 0.3 | - | 66.7% | 33.3% | - | - |
| | 0.4 | 100% | - | - | - | - |
| 90° | 0.1 | - | - | 100% | - | - |
| | 0.2 | - | 100% | - | - | - |
| | 0.3 | - | 100% | - | - | - |
| | 0.4 | 100% | - | - | - | - |
